# The Psychometric Properties of Autism Mental Status Examination (AMSE) in Turkish Sample

**DOI:** 10.1007/s10803-025-06761-8

**Published:** 2025-03-04

**Authors:** Yavuz Meral, Alperen Bıkmazer, Abdurrahman Cahid Örengül, Süleyman Çakıroğlu, Esra Altınbilek, Fulya Bakır, Bilgihan Bıkmazer, Ayman Saleh, Vahdet Görmez

**Affiliations:** 1 Istanbul Medeniyet University, School of Medicine, Department of Child and Adolescent Psychiatry, Istanbul, Türkiye; 2 Istanbul University, Istanbul Faculty of Medicine, Department of Child and Adolescent Psychiatry, Istanbul, Türkiye; 3 Altınbaş University, School of Medicine, Child and Adolescent Psychiatry, Istanbul, Türkiye; 4 Göztepe Prof. Dr. Süleyman Yalçın City Hospital, Istanbul, Türkiye; 5 Marmara University Training and Research Hospital, Pediatric Neurology Clinic, Istanbul, Türkiye; 6 George Washington University, Children’s National Hospital, Psychiatry Department, Washington, USA; 7 Hamad Bin Khalifa University, College of Islamic Studies, Doha, Qatar

**Keywords:** Autism spectrum disorder, Autism mental status examination, Reliability, Validity, Early diagnosis

## Abstract

Autism Spectrum Disorder (ASD) is a prevalent neurodevelopmental disorder, and early diagnosis plays a pivotal role in prognosis and management. This study aims to examine the psychometric properties of the Autism Mental Status Exam (AMSE), a tool that shows great promise in terms of clinical utility, within the Turkish population. This study conducted in a cohort of 307 Turkish children aged 17 to 120 months with suspected ASD. Participants underwent a multidisciplinary assessment based on DSM-5 criteria for diagnosis and were categorized into ASD and non-ASD groups. Subsequently, the research team conducted blinded administrations of the AMSE and Childhood Autism Rating Scale (CARS). Additionally, a subset of 61 children underwent retesting for AMSE and CARS after three weeks for temporal stability. The results revealed an optimal cut-off score of 4 for AMSE, yielding sensitivity and specificity rates of 84% and 97%, respectively. Internal consistency, indicated by a Cronbach’s alpha of 0.80, was very good. The test-retest reliability, assessed using the Intraclass Correlation Coefficient (ICC), was excellent (*ICC* = 0.959). The inter-rater reliability also showed excellent agreement (*ICC* = 0.997). Furthermore, a significant correlation was observed between the AMSE and CARS scores (*r* = 0.94, *p* < 0.001). Notably, the AMSE scores were significantly different between the ASD and non-ASD groups (*p* < 0.001) with a large effect size (*Cohen’s d* = 1.40). The findings of this study underscore the utility of AMSE as a valid and reliable tool for Turkish children with robust psychometric properties.

## Introduction

Autism Spectrum Disorder (ASD) is a common neurodevelopmental condition that impacts social functioning, emotions, and behaviors, with lifelong implications (American Psychiatric Association, 2013). The global prevalence of ASD in children is estimated to be 1% ( Zeidan et al., 2022), and evidence points to a growing trend in autism diagnoses (Talantseva et al., 2023). According to a 2020 surveillance conducted by CDS, the prevalence of ASD at 8 years was reported to be 2.76%, or approximately 1 in every 36 children (Maenner et al., 2023). Factors contributing to this rise may include heightened awareness, wider availability of diagnostic tools, improved access to healthcare services, and a broader diagnostic definition of autism (Zeidan et al., 2022). The diverse nature of ASD, along with varying developmental stages, gender differences, comorbidities, and intellectual functioning levels, can make diagnosis challenging (Daniels & Mandell, 2014). Early diagnosis is essential for achieving effective treatment outcomes (Corsello, 2005; Estes et al., 2015). However, in a nationally representative sample of children with ASD in the United States, the average time between referral and diagnosis was found to be approximately 2.7 years (Zuckerman et al., 2015). This delay in diagnosis significantly hinders early intervention during critical developmental periods, leading to substantial setbacks (Zwaigenbaum et al., 2015).

In Türkiye, the healthcare system operates on a tiered structure where general practitioners or pediatricians typically refer suspected ASD cases to child and adolescent psychiatrists. This referral process is integrated into the Ministry of Health’s Autism Screening Program, which employs the Modified Checklist for Autism in Toddlers (M-CHAT) to identify children requiring further evaluation (Dursun et al., 2022). Parents also have the option to directly schedule appointments with child and adolescent psychiatrists, bypassing the referral process. However, the limited number of specialists relative to the population (OECD, 2025) and socio-demographic disadvantages lead to delays in evaluation and diagnosis (Yaylaci & Guller, 2023). In addition, policies aimed at reducing wait times results in shorter evaluation sessions in clinics, typically averaging 20 min (Republic of Türkiye Ministry of Health, 2025). This time constraint limits the feasibility of employing comprehensive diagnostic guidelines and tools for ASD that require extended assessment durations. Consequently, clinicians primarily rely on widely accepted frameworks, such as the Diagnostic and Statistical Manual of Mental Disorders, Fifth Edition (DSM-5) and the International Classification of Diseases, 11th Revision (ICD-11), to guide the diagnostic process in clinical practice, with tools like the Schedule for Affective Disorders and Schizophrenia for School-Aged Children: Present and Lifetime Version (K-SADS-PL DSM-5) being utilized predominantly in research settings.

The current gold standard approach to autism diagnosis is the tiered application of semi-structured methods, such as ADOS-2 (Lord et al., 2012) and ADI-R (Rutter et al., 2003), supplemented by clinical interviews based on DSM-5. However, the practicality and ease of use of these instruments are significantly limited in Türkiye by many factors including high costs, time-consuming procedures, licensing restrictions, and extensive training requirements. Moreover, these costly diagnostic tools are not necessary for every case, especially in clinical settings. Parent-based observation tools for autism assessment and screening, such as Modified Checklist for Autism in Toddlers (M-CHAT) (Kondolot et al., 2016; Robins et al., 2001), Autism Behavior Checklist (ABC) (Krug et al., 1980; Ozdemir et al., 2013), Autism Spectrum Screening Questionnaire (ASSQ) (Ehlers et al., 1999; Kose et al., 2015) and Social Communication Questionnaire (SCQ) (Avcil et al., 2015; Berument et al., 1999), are valid and reliable tools for the Turkish population, though self-reports are known to have limitations, such as over-reporting as well as under-reporting (Johnson et al., 2009). These screening tools are frequently utilized at all levels of care for both clinical evaluation and research purposes; however, by their nature, they are not designed to serve as a diagnostic tool.

Recently, interactive screening tools like the Rapid Interactive Screening Test for Autism (RITA) (Choueiri & Wagner, 2015) have been validated in Turkish samples as second-step screening instruments to better identify high risk populations (Kadak et al., 2024). However, the RITA is designed for use by non-experts, rather than clinicians, to facilitate triage. The Childhood Autism Rating Scale (CARS) is widely used by specialists to aid ASD diagnosis (Gassaloğlu et al., 2016). Initially developed to distinguish between developmental delay, such as intellectual disabilities, and ASD, CARS is also used for research purposes. Clinicians employ CARS to evaluate various areas in addition to specific ASD-related items (Schopler et al., 1980). However, compared to the AMSE, which is seamlessly integrated into clinical observation and requires approximately 5–10 min for scoring, the CARS demands significantly more time to be used effectively, typically ranging from 20 to 30 min depending on the clinician’s level of expertise (Kılınç et al., 2019). The shorter administration time may be attributed to AMSE’s fewer items, simplified Likert scale, reliance on rapid observational assessment, and more practical scoring system compared to CARS. In addition to these challenges regarding ASD diagnosis, the competence and experience of healthcare professionals other than child and adolescent psychiatrists in Türkiye are also a subject of concern. Although arguable, (Tamur & Şen Celasin, 2022) some studies proposed that primary care physicians often lack the expertise and face significant challenges in effectively managing cases with ASD (Alpdoğan et al., 2023).

The AMSE was developed by Grodberg et al. (2012) through collaborative efforts involving child psychiatrists, behavioral pediatricians, and child neurologists as a diagnostic assessment tool based on observational cues for the core symptoms of ASD, including social interaction, communication skills, and behavioral patterns (Grodberg et al., 2012). Unlike parent-report screening tools such as the M-CHAT, ABC, ASSQ or SCQ, which rely on caregiver observations, the AMSE is a clinician-administered tool that examines both direct behavioral symptoms and caregiver reports, providing a structured, real-time assessment of ASD symptoms. Additionally, the AMSE helps standardize the clinical observation process, ensuring a more consistent and objective evaluation across different clinical settings. The AMSE is a brief, easy-to-use, and free observational tool that does not add to clinicians’ workload, supporting ASD diagnosis. In addition to its original development studies in the USA (Grodberg et al., 2012, 2014, 2016), the validity of AMSE has been demonstrated in several countries, including France (Pagnier & Chaste, 2022), Norway (Øien et al., 2018, 2020), Sweden (Cederlund, 2019), Chile (Irarrázaval et al., 2023), China (Yang et al., 2023), and Brazil (Galdino et al., 2020). AMSE has also been successful in achieving diagnostic differentiation with high specificity and sensitivity for developmental delay (Betz et al., 2019), ADHD (Øien et al., 2020), and anxiety comorbidities (Arnold et al., 2016) both in individuals with and without ASD. The psychometric properties of AMSE have been examined in different languages and populations; however, no studies have previously investigated its psychometric properties in Türkiye. This study is the first to assess its validity, reliability, and cut-off values in the Turkish population.

In Türkiye, there is a lack of diagnostic observation tools that standardize ASD examinations in clinical practice- tools that are easy to apply, low-cost, and time-efficient. Globally, there is a need for quick, practical observation tools that integrate feedback without increasing the clinician’s workload in the diagnosis of ASD. This study aims to examine the psychometric properties of AMSE, including its validity, reliability, interrater reliability, and cut-off scores, in Turkish children with suspected ASD.

## Methods

### Participants

The study was conducted at Istanbul Medeniyet University, Child and Adolescent Psychiatry Clinic for Neurodevelopmental Delay (IMU CND). The sample consisted of 307 participants including 76 girls and 231 boys, aged 17 to 120 months (*M* = 35.5 ± 19.04). The IMU CND is a unit where children identified as suspected ASD by primary healthcare institutions and pediatric clinics, under the ASD screening and follow-up program conducted by the Ministry of Health of the Republic of Türkiye, undergo advanced evaluation, diagnosis, and treatment management. The multidisciplinary team at the clinic is led by a senior child and adolescent psychiatrist (V.G.), a faculty member at child and adolescent psychiatry with over 10 years of professional experience and certified in the utilization of the ADOS. The child development specialist has nearly 3 years of expertise in conducting developmental evaluations for ASD. The clinical psychologist (E.A.), with approximately 15 years of experience, specializes in tracking the developmental progress, and behavioral problems of children with ASD and administering standardized assessment tools including CARS. Furthermore, the child neurologist (B.B.) provides consultations on cases requiring advanced neurological evaluation. At this unit ASD diagnoses were made based on DSM-5 clinical evaluations, and treatment plans were managed accordingly.

Participants in the study were children referred to the IMU CND between July 2019 and January 2020 who agreed to participate. Exclusion criteria included sensory impairments, current or past neurological, metabolic, or genetic syndromes, and refusal to participate in the study. The required sample for inter-rater reliability consisted of the first 30 participants in the study, while the sample for the test-retest procedure was randomly choosen from the entire sample, ensuring representativeness of both ASD and non-ASD groups. Based on the two-observer model and previously reported excellent inter-rater intraclass correlation coefficient (ICC) values (Grodberg et al., 2012; Arnold et al., 2016), the minimum sample size was determined to be 25 participants with a 90% power (Bujang & Baharum, 2017). Since no prior reference for test-retest reliability of AMSE existed in the literature, the expected ICC was set at 0.6. When ICC is estimated at 0.6, a sample size of 15 cases ensures 80% power, while 20 cases ensure 90% power (Bujang & Baharum, 2017). To ensure the inclusion of at least 20 ASD cases and to adequately represent the overall sample, we calculated a minimum of 60 subjects regarding the ASD to non-ASD ratio. To account for potential dropouts, 66 cases were randomized for the retest phase, of which 61 participants included in the test-retest assessment. Among the these children, 20 were in the ASD group, and 41 were in the non-ASD group.

Legal representatives of the children were informed about the study’s content and objectives, and both verbal and written informed consent were obtained. The study was approved by the Istanbul Medeniyet University Goztepe Training and Research Hospital Ethics Committee (approval date and number: 15/5/2019 − 2018/0342).

### Measurements

#### Sociodemographic Form

Participants’ age, sex, and family financial status were collected using a researcher-designed sociodemographic form.

#### Autism Mental Status Examination (AMSE)

The AMSE is a clinical assessment tool conceptualized according to the DSM criteria for ASD to assist in identifying individuals on the spectrum. It consists of eight items assessing eye contact, interest in others, pointing skills, language abilities, pragmatics, repetitive behaviors, preoccupations, and unusual sensitivities. Each item is scored from 0 to 2, resulting in a total score ranging from 0 to 14. The scoring for the first three items of the AMSE—eye contact, interest in others, and pointing skills—is based on clinician observation during naturalistic interactions throughout the assessment. These items are evaluated without requiring specific interventions, relying instead on the individual’s spontaneous behaviors. For instance, pointing skills may be assessed through spontaneous pointing or by prompting the individual to locate an object in the room. The item assessing pragmatic language evaluates features such as pedantic speech, difficulties with small talk, unclear answers, echolalia, or atypical intonation patterns. For verbal individuals, the presence of any of these features is scored as 2 if directly observed during the assessment or as 1 if not observed but reliably reported by caregivers. Notably, this item is only applicable if item 4 (Language) is scored as 0, indicating the presence of verbal communication skills. If item 4 is scored as 1 or 2, reflecting limited or absent language abilities, this item is automatically skipped. The remaining items assess repetitive behaviors and stereotypies, such as hand flapping, echolalia, or rigid behavioral patterns, based on observation or caregiver reports. AMSE also evaluate intense, atypical preoccupations that dominate the individual’s focus within the past two weeks and unusual sensory sensitivities, including heightened or diminished reactions to noise, texture, or pain. Higher scores indicate more pronounced autistic symptoms. See Appendix A for the AMSE scoring list summary.

The AMSE is easy to administer, free of charge, and does not add a significant burden to clinical practice, contributing to its broad utility. Previous studies conducted in both child and adult populations have demonstrated robust psychometric properties and have been translated into numerous languages. The psychometric properties of the AMSE indicate high sensitivity and specificity along with acceptable internal consistency. The diagnostic adequacy of this tool has been evaluated against the DSM-5 (Galdino et al., 2020; Grodberg et al., 2016; Yang et al., 2023), ADOS (Grodberg et al., 2012, 2014), ADOS-2 (Pagnier & Chaste, 2022; Øien et al., 2018; Irarrázaval et al., 2023), ADI-R (Pagnier & Chaste, 2022; Øien et al., 2020), ICD-10, and DISCO-11 (Cederlund, 2019) across populations ranging from 18 months to adulthood, yielding promising results. Preliminary test performance data from toddlers to adults indicated the solid psychometric properties of the AMSE of various languages. Table 1 provides a summary of the psychometric properties of AMSE reported in previous studies. Additional information on AMSE usage and scoring, including a free online curriculum with video-simulated cases, is available on the website (http://autismmentalstatusexam.com/) (Grodberg et al., 2012).

**Table 1:** Psychometric properties of the AMSE in previous studies

Study	Age	Cut-off Value	Sensitivity	Specificity	Cronbach’s Alpha
Grodberg et al. (2012) (USA)	18-months through 38-years (*M* = 12.7, *SD* = ± 8.05)	5	0.94	0.81	0.72
Grodberg et al. (2014) (USA)	Fluent adults 18 through 45 years (*M* = 28.90, *SD* = ± 8.29)	5	0.93	0.91	Not reported
Grodberg et al. (2016) (USA)	18 months to 60 months (*M* = 41.1 *SD* = ± 12.5) (high-risk non-verbal ASD)	6	0.94	1.0	Not reported
Arnold et al. (2016) (USA)	12 months to 144 months (*M* = 79.44, *SD* = ± 43.25)	5	Not reported	Not reported	0.64
Øien et al. (2018) (Norway)	*M* = 5.74 years, *SD* = ± 2.88 years	5	Not reported	Not reported	Not reported
Betz et al. (2019) (USA)	18 to 68 months (*M* = 37.9 months, *SD* = ± 15.2)	5	0.812	0.905	Not reported
Cederlund (2019) (Sweeden)	Preschool children (*M* = 54.4 months, *SD* = ± 15.8 months)	7	0.75	0.78	Not reported
Øien et al. (2020) (Norway)	36 months to 12 years (*M* = 7.38 years, *SD* = ± 2.44 years)	5	0.83	0.90	Not reported
Galdino et al. (2020) (Brazil)	3 to 18 years (*M* = 9.1 years *SD* = ± 3.4 years)	4	0.91	0.98	0.74
Pagnier and Chaste (2022) (France)	13 months to 17 years and 9 months (*M* = 59.0 months, *SD* = ± 35.3 months)	5	0.95	0.67	0.67
Yang et al. (2023) (China)	2 to 11 years (*M* = 5.23 years, *SD* = ± 2.30 years)	6	0.98	0.87	0.65
Irarrázaval et al. (2023) (Chile)	15 months to 17 years (*M* = 6.4 years, *SD* = ± 3.6 years)	6	0.79	0.92	0.61

#### Childhood Autism Rating Scale (CARS)

The CARS is a scale in which symptoms of autism are observed and evaluated by clinicians based on 15 items. It was developed to differentiate between ASD and developmental disabilities (Schopler et al., 1980). Each item is scored from 1 to 4, resulting in a total score ranging from 15 to 60. The CARS categorizes ASD into varying degrees of severity, including classifications such as “non-autistic,” “mild to moderate,” and “severe.” According to a Turkish validity and reliability study conducted with a Turkish sample, the Cronbach’s alpha value of the scale was 0.95, indicating high internal consistency. The test-retest reliability for the total score was determined to be *r* = 0.98 (*p* < 0.01), and the inter-rater reliability was found to be *r* = 0.97 (*p* < 0.01). The cut-off score was determined to be 29.5. The psychometric properties of the scale demonstrate that the CARS is a valid and reliable measurement tool in the Turkish language (Gassaloğlu et al., 2016).

### Procedure

The AMSE was first translated into Turkish by two senior child and adolescent psychiatric specialists (V.G. and A.C.Ö.). A bilingual medical student, unaware of the questionnaire, back-translated it into English. One of the authors (V.G.) reviewed the back-translation. Subsequently, final adjustments were made in consultation with the team of Dr. Grodberg.

The cases included in the study were evaluated for an ASD diagnosis based on DSM-5 criteria through a comprehensive clinical assessment conducted by the IMU CND team, led by a senior child and adolescent psychiatrist with expertise in ASD (V.G.). The evaluation process included a 45-minute detailed developmental, psychiatric, and medical history assessment, followed by a 15-minute free-play interaction with the child. If available, recorded videos or interaction footage from the child’s natural environment, provided by parents, were also reviewed. In line with routine practice, parent-rated tools such as the M-CHAT, ABC, or SCQ were utilized whenever available. Additionally, standardized developmental assessments, including the Denver II Developmental Screening Test (Yalaz et al., 2009) and Ankara Developmental Screening Inventory (Sezgin, 2011), were administered by a child development specialist. Within the same session of the diagnostic evaluation process, the child and adolescent psychiatrist conducting the diagnostic interviews (V.G.) also administered the AMSE to the first 30 cases to assess inter-rater reliability. Although the diagnostic procedure reflects the routine practice at IMU CND, in this study, the CARS was independently administered by a clinical psychologist in a blinded manner after the initial assessment. Based on these evaluation results, the patients were divided into two groups: those with and without ASD. Subsequently, the AMSE was administered independently and in a blinded manner by a second senior child and adolescent psychiatrist (A.B.), a faculty member with approximately eight years of professional experience in child and adolescent psychiatry. AMSE was successfully re-administered to 61 participants at the third week by a second child and adolescent psychiatrist (V.G.) to evaluate test-retest reliability.

### Data Analysis

Descriptive statistics were used to assess the quality and discriminative capacity of the items within the AMSE, along with the total score of the CARS. Cronbach’s alpha was employed to ascertain the homogeneity and internal consistency of the AMSE items, while inter-rater reliability was evaluated using the ICC. Test-retest reliability was examined by analyzing the data obtained from the initial and three-week follow-up administrations of AMSE using Pearson correlation analysis and the intraclass correlation coefficient (ICC, two-way mixed-effects model). Sensitivity, specificity, positive predictive value (PPV), negative predictive value (NPV), and receiver operating characteristic (ROC) curve cut-off points were computed to gauge the sensitivity of AMSE. Criterion validity was established via correlations between different scales. Exploratory factor analysis (EFA) was conducted using principal axis factoring with varimax rotation, while confirmatory factor analysis (CFA) was performed using the maximum likelihood (ML) method to validate the underlying factor structure of the AMSE. Independent sample t-tests were performed to examine age and AMSE scores differences between groups, comparing those with ASD to those without. The relationship between age and AMSE scores was analyzed using Pearson correlation analysis. To control for the effect of age in the comparison between the ASD and non-ASD groups, ANCOVA was performed. Cohen’s d is used to determine effect size. Test-retest reliability, inter-rater reliability, Cronbach’s alpha, and the correlation between AMSE and CARS scores were further analyzed separately for the ASD and non-ASD groups. A significance level of *α* = 0.05 was adopted for statistical analyses. Data analysis was performed using IBM SPSS Statistics 23 software package.

## Results

### Descriptive Statistics

A total of 307 children aged between 17 and 120 months (*M* = 35.5 ± 19.04) were included in the study. Seventysix of the children were girls and 231 were boys. ASD was diagnosed in 33% (*n* = 101) of children included in the study. Among the 101 patients diagnosed with ASD, 76 were male and 25 were female, resulting in a male-to-female ratio of 3:1.

### Item Analysis, Internal Consistency, Test-Retest Reliability, Intraclass and Inter-Rater Correlation Reliability

Item analysis was used to evaluate the quality and discrimination of the scale items. According to the item analysis results, the item-rest correlation values ranged between 0.07 and 0.77 (Table 2). The internal consistency of the scale was tested using Cronbach’s alpha. The total Cronbach’s alpha for the scale was 0.80.

**Table 2:** Item analysis of AMSE

Item	Mean	SD	Item-Rest correlation	If the item dropped Cronbach’s alpha
AMSE1	0.329	0.595	0.6950	0.762
AMSE2	0.431	0.666	0.7745	0.746
AMSE3	0.385	0.694	0.7060	0.757
AMSE4	0.944	0.557	0.2863	0.818
AMSE5	0.141	0.497	0.0767	0.838
AMSE6	0.497	0.829	0.6290	0.772
AMSE7	0.220	0.575	0.5454	0.785
AMSE8	0.158	0.461	0.4769	0.795

To evaluate test-retest and ICC, the instrument was administered to 61 randomly selected children twice at an interval of three weeks. The ICC of the AMSE total score was 0.959 (*p* < 0.001) indicating strong temporal stability over time. The inter-rater reliability of the total AMSE score was excellent, with an ICC of 0.997. The reliability of the eight individual items were as follows: Eye Contact (*ICC* = 0.962), Interest in Others (*ICC* = 0.981), Pointing Skills (*ICC* = 0.995), Language (*ICC* = 0.981), Language Pragmatics (*ICC* = 1.00), Repetitive Behaviors (*ICC* = 1.00), Unusual or Extensive Preoccupations (*ICC* = 0.993), and Unusual Sensitivities (*ICC* = 0.981). The correlation values for each item and total AMSE score are shown in Table 3.

**Table 3:** Correlations between the total AMSE score and each item

		AMSE1	AMSE2	AMSE3	AMSE4	AMSE5	AMSE6	AMSE7	AMSE8	AMSE-total
AMSE1	*r*	—								
	*p*	—								
AMSE2	*r*	0.732	—							
	*p*	< 0.001	—							
AMSE3	*r*	0.684	0.746	—						
	*p*	< 0.001	< 0.001	—						
AMSE4	*r*	0.365	0.385	0.457	—					
	*p*	< 0.001	< 0.001	< 0.001	—					
AMSE5	*r*	0.032	0.094	-0.082	-0.400	—				
	*p*	**0.580**	**0.101**	**0.155**	< 0.001	—				
AMSE6	*r*	0.458	0.538	0.493	0.125	0.318	—			
	*p*	< 0.001	< 0.001	< 0.001	0.030	< 0.001	—			
AMSE7	*r*	0.395	0.457	0.407	0.142	0.225	0.462	—		
	*p*	< 0.001	< 0.001	< 0.001	0.013	< 0.001	< 0.001	—		
AMSE8	*r*	0.340	0.358	0.335	0.201	0.104	0.442	0.391	—	
	*p*	< 0.001	< 0.001	< 0.001	< 0.001	0.071	< 0.001	< 0.001	—	
AMSE-total	*r*	0.775	0.846	0.801	0.432	0.237	0.770	0.672	0.584	—
	*p*	< 0.001	< 0.001	< 0.001	< 0.001	< 0.001	< 0.001	< 0.001	< 0.001	—

### Criterion Validity

The criterion validity of the AMSE was assessed using the CARS, which has been validated and is reliable in Turkish children and is frequently used (Gassaloğlu et al., 2016). A positive, strong, and significant correlation was found between the AMSE and CARS total scores (*r* = 0.94; *p* < 0.001).

### Structural Validity of the Scale

The sample of 307 participants in our study was deemed sufficient for conducting EFA and CFA analyses. Tabachnick and Fidell (2013) recommends a minimum sample size of 300 for EFA, although a sample size of 150–200 may be considered adequate when factor loadings are 0.6 or higher. Similar guidelines apply to CFA, where the item count × 10 rule is also suggested as a criterion for determining sample adequacy (Tabachnick & Fidell, 2013).

#### Results of Exploratory Factor Analysis

EFA revealed a two-factor structure, consistent with the theoretical subdimensions of AMSE (Grodberg et al., 2012). The Kaiser-Meyer-Olkin (KMO) measure was 0.82, and Bartlett’s Test of Sphericity was significant (*χ²*(28) = 450.0, *p* < 0.001), confirming the adequacy of the data for factor analysis. The first factor, Social Communication and Language, accounted for 45% of the variance and included items related to Eye Contact (0.78), Interest in Others (0.80), Pointing Skills (0.75), Language (0.65), and Language Pragmatics (0.60). The second factor, Repetitive Behaviors and Sensory Differences, explained 25% of the variance and comprised items assessing Repetitive Behaviors (0.82), Unusual Preoccupations (0.77), and Unusual Sensitivities (0.70). Factor loadings varied across items, with higher loadings observed for core symptoms (e.g., Interest in Others = 0.80, Repetitive Behaviors = 0.82) and slightly lower but still substantial loadings for language-related items (e.g., Language Pragmatics = 0.60). Together, these two factors accounted for 70% of the total variance, supporting the proposed AMSE subdimensions. (Table 4)

**Table 4:** Exploratory factor analysis for AMSE items (*n* = 307)

Item	Factor 1: Social Communication and Language	Factor 2: Repetitive Behaviors and Sensory Differences
Eye Contact	0.78	—
Interest in Others	0.80	—
Pointing Skills	0.75	—
Language	0.65	—
Language Pragmatics	0.60 *(Applicable when language is present)*	—
Repetitive Behaviors	—	0.82
Unusual or Extensive Preoccupations	—	0.77
Unusual Sensitivities	—	0.70
Explained Variance (%)	45.0%	25.0%
Total Explained Variance (%)	**70.0%**	

**Notes**: Kaiser-Meyer-Olkin (KMO) Measure: 0.82, Bartlett’s Test of Sphericity: *χ²*(28) = 450.0, *p* < 0.001. Factor loadings below 0.40 were suppressed for clarity

#### Results of Confirmatory Factor Analysis

Building upon the findings from the EFA, a two-factor model was tested using CFA. The model fit indices indicated a good fit to the data: *χ²*(19) = 40.5, *p* < 0.01, *RMSEA* = 0.06, *CFI* = 0.97, and *SRMR* = 0.04. These values align with established criteria for acceptable model fit (*CFI* > 0.95, *RMSEA* < 0.08, *SRMR* < 0.05), supporting the validity of the proposed two-factor structure. Additionally, all factor loadings ranged from 0.60 to 0.85 and were found to be statistically significant (*p* < 0.001), demonstrating that each item made a substantial contribution to its respective factor. These findings provide strong empirical support for the structural integrity of the AMSE’s two-factor model, reinforcing the distinction between social-communicative and behavioral-sensory dimensions.

### Sensitivity, Specificity, Positive Predictive Value (PPV), Negative Predictive Value (NPV) and Cut-Off Point

**Fig. 1:**
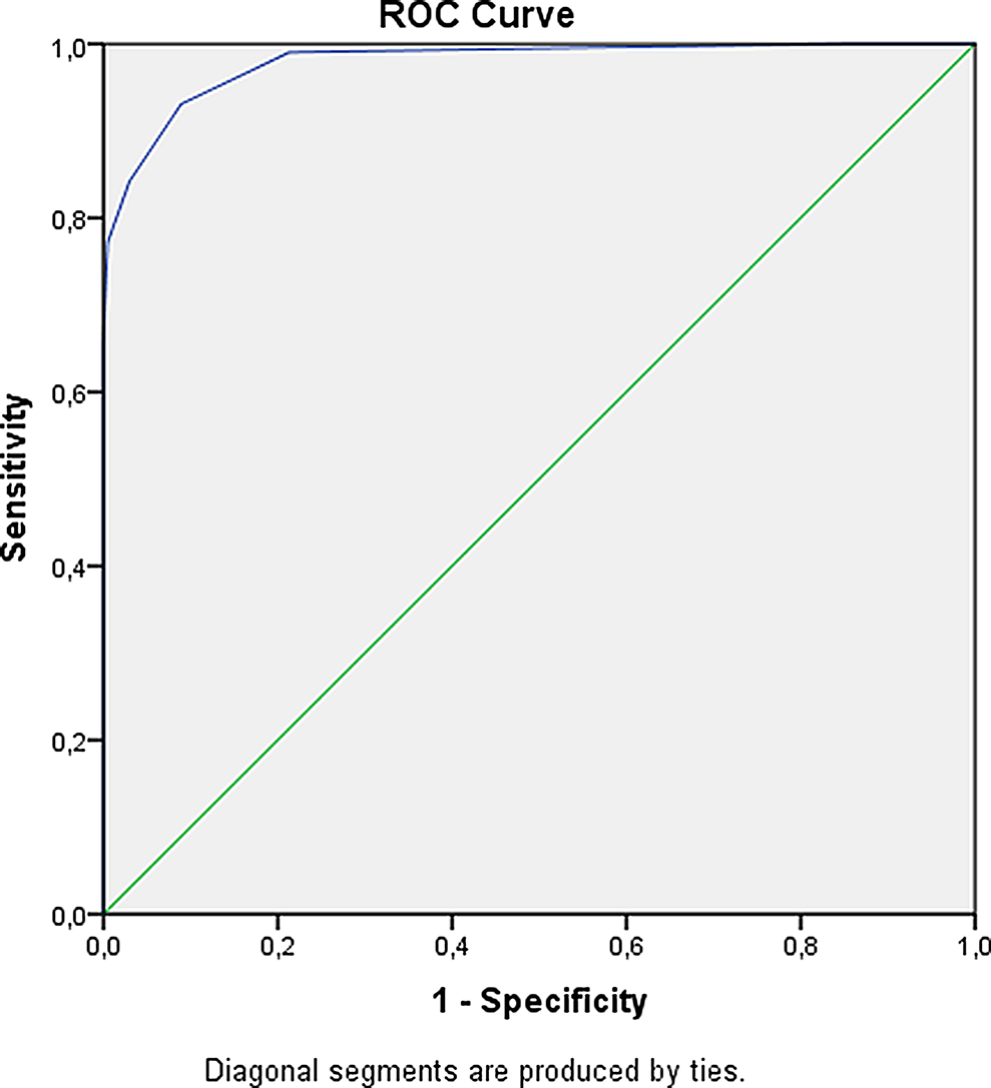
ROC curve illustrating model performance and the sensitivity-specificity relationships for AMSE

The sensitivity, specificity, positive predictive value (PPV), negative predictive value (NPV), and cut-off point for AMSE were calculated using the ROC curve. The curve illustrates the model’s ability to distinguish between positive and negative classes at different cut-off values. The results of the ROC analysis showed that the residual under the curve (UAC) was significant at 0.979. With a cut-off score of 4, the sensitivity and specificity were 84.16% and 97.03%, respectively; with a cut-off score of 5, the sensitivity and specificity were 77.23% and 99.5%, respectively. When the optimal cut-off was set at 4, the PPV was found to be 93.41% and the NPV 92.45%. At a cut-off of 6, the PPV reached 100%, while at a cut-off of 1, the NPV reached 100%. The current findings indicate that a score of 4 represents the optimal cut-off value. The results are presented in Table 5. The ROC curve is shown in Fig. 1.

**Table 5:** Sensitivity, specificity, PPV, NPV and cut-off point of AMSE

Cut-off	Sensitivity (%)	Specificity (%)	PPV (%)	NPV (%)	Youden’s index	AUC	Metric Score
0	100%	0%	33.33%	NaN%	0.0000	0.979	1.00
1	100%	14.85%	37%	100%	0.1485	0.979	1.15
2	99.01%	78.71%	69.93%	99.38%	0.7772	0.979	1.78
3	93.07%	91.09%	83.93%	96.34%	0.8416	0.979	1.84
4	84.16%	97.03%	93.41%	92.45%	0.8119	0.979	1.81
5	77.23%	99.5%	98.73%	89.73%	0.7673	0.979	1.77
6	65.35%	100%	100%	85.23%	0.6535	0.979	1.65
7	52.48%	100%	100%	80.8%	0.5248	0.979	1.52
8	43.56%	100%	100%	77.99%	0.4356	0.979	1.44
9	31.68%	100%	100%	74.54%	0.3168	0.979	1.32
10	16.83%	100%	100%	70.63%	0.1683	0.979	1.17
11	9.9%	100%	100%	68.94%	0.0990	0.979	1.10
12	6.93%	100%	100%	68.24%	0.0693	0.979	1.07
13	1.98%	100%	100%	67.11%	0.0198	0.979	1.02

### Comparison of AMSE Scores of Groups with and without Autism Diagnosis

The mean AMSE total scores differed significantly between the ASD and non-ASD groups. The ASD group (*M* = 6.82, *SD* = 2.88, *n* = 101) had substantially higher scores compared to the non-ASD group (*M* = 1.19, *SD* = 0.803, *n* = 202). In the overall sample, there was a weak and non-significant relationship between age and AMSE scores (*r* = 0.08, *p* = 0.10). No significant difference in age was found between the ASD and non-ASD groups (*p* = 0.42). ANCOVA was conducted to examine the effect of diagnostic group on AMSE total scores, while controlling for age as a covariate. The results indicated a significant main effect of diagnostic group on AMSE scores, *F*(1, 300) = 150.00, *p* < 0.001, partial *η²* = 0.33, suggesting a large effect size and substantial differentiation between ASD and non-ASD groups. In contrast, age did not have a significant effect on AMSE scores, *F*(1, 300) = 2.10, *p* = 0.15, partial *η²* = 0.007, indicating that AMSE scores were not influenced by age. The effect size was found to be very large (*Cohen’s d* = 1.40).

### Comparison of Psychometric Properties of the AMSE in ASD and Non-ASD Groups

Both the ASD and non-ASD groups demonstrated high test-retest and inter-rater reliability, with ICC exceeding 0.95. Internal consistency, assessed using Cronbach’s alpha, was 0.79 in the ASD group and 0.81 in the non-ASD group, suggesting that the scale exhibits adequate reliability in terms of item consistency across groups. When analyzed separately by group, the correlation remained strong in the ASD group (*r* = 0.88) but was weaker in the non-ASD group (*r* = 0.45). Table 6 provides a detailed comparison of these findings.

**Table 6:** Psychometric properties of the AMSE in ASD and non-ASD groups

Psychometric Property	ASD Group (*n* = 101)	Non-ASD Group (*n* = 206)
Test-Retest Reliability	*r* = 0.91, *p* < 0.001	*r* = 0.92, *p* < 0.001
Intraclass Correlation Coefficient (ICC) for Test-Retest	*ICC* = 0.955, *p* < 0.001	*ICC* = 0.960, *p* < 0.001
Inter-Rater Reliability (Total Score ICC)	*ICC* = 0.995, *p* < 0.001	*ICC* = 0.998, *p* < 0.001
Internal Consistency (Cronbach’s Alpha)	*α* = 0.79	*α* = 0.81
Correlation Between AMSE and CARS Scores	*r* = 0.88, *p* < 0.001	*r* = 0.45, *p* = 0.002

## Discussion

AMSE is a valuable diagnostic aid that can incorporate caregiver reports along with observation-based measurements without adding an extra burden to clinical practice (Grodberg et al., 2012). The results of the present study demonstrated that the AMSE can effectively differentiate ASD symptoms with high sensitivity and specificity, particularly when utilizing a cut-off score of 4. It showed excellent inter-rater reliability and temporal stability. This study is the first to examine the psychometric properties of the AMSE using a Turkish sample, establishing it as a valid and reliable diagnostic observation tool.

When the results of multidisciplinary evaluations based on the DSM-5 criteria were considered as the comparison standard for diagnosis, the predictive value was found to be 4, with a sensitivity of 84.16% and specificity of 97.03%. When this value was 5, the sensitivity decreased to 77.23%, but the specificity increased to 99.5%. Similarly, Galdino et al. (2020) found that a predictive value of 4 was optimal for a Brazilian sample of 260 children, reporting a sensitivity of 91% and specificity of 98% (Galdino et al., 2020). In addition to the numbers being relatively close to each other, notable methodological similarities include the diagnostic processes being similar and the control groups consisting of clinical samples. However, the age range in the Brazilian sample also included adolescents older than those in our population (*M* = 9.1; *SD* = ± 3.4). Although the majority of previous studies reported a cut-off value of 5 (Betz et al., 2019; Grodberg et al., 2012, 2014; Øien et al., 2020; Pagnier & Chaste, 2022), there were also studies that reported cut-off values of 6 (Grodberg et al., 2016; Irarrázaval et al., 2023; Yang et al., 2023) and 7 (Cederlund, 2019). The variation in cut-off scores across different studies does not appear to be solely attributable to the age characteristics of the sample. For example, Grodberg et al. (2016) reported an optimal cut-off value of 6 in a population with a mean age of 41.1 months (± 12.5 months) which is comparable to the mean age in our study of 35.5 months (± 19.04 months) (Grodberg et al., 2016). Similarly, a study conducted with a preschool sample in Sweden identifed an optimal cut-off value of 7 (Cederlund, 2019). Pagnier and Chaste (2022) argued that the optimal predictive value varies across different age and sex groups, with these parameters significantly influencing cut-off scores, whereas adaptive behaviors showed no such effect. Their study revealed age-related variations in optimal cut-off values, with a cut-off of 6 (sensitivity: 0.92, specificity: 0.67) for participants aged 0 to 36 months, a cut-off of 5 (sensitivity: 0.98, specificity: 0.62) for those aged 3 to 6 years, and a cut-off of 6 (sensitivity: 0.86, specificity: 0.67) for participants aged 6 years and older highlighting the absence of a linear variability relationship specific to age. They further suggested that a specific range between scores of 3 and 9 might be more suitable for in-depth examination and guidance. The authors proposed that cut-off scores could support expert clinicians during clinical interviews and facilitate the direct referral of cases within certain ranges to tertiary healthcare centers by primary care health professionals, thereby reducing the need for additional testing and preventing diagnostic delays (Pagnier & Chaste, 2022). Irarrázaval et al. (2023) argued that the diagnostic capacity of AMSE is more effective in younger individuals and those with lower language abilities, despite the cut-off value remaining consistent across different verbal skills (Irarrázaval et al., 2023). Although definitive conclusions cannot be drawn from the current data, discrepancies in specifity, sensitivity and optimal cut-off across studies may be attributed to variations in sex, sample size, sample characteristics (including comorbidities), diagnostic instruments and scales used, as well as the level of expertise among practitioners. This study consists of a sample of participants identified as suspected ASD cases, the majority of whom referred due to neurodevelopmental complaints such as speech delay. While this aligns with the AMSE’s intended target population, it should be noted that the the psychometric properties of AMSE may have been influenced by these parameters excluded from the scope of this study. Since this study was conducted with a sample consisting of individuals suspected of having ASD, caution is warranted when attempting to generalize the findings to the entire population. Collectively, these findings indicate solid sensitivity and specificity values in the Turkish sample. Well-designed future studies with rigorous control of confounding factors are needed to clarify variations in the results.

The internal consistency of the AMSE was very good (Cronbach’s alpha = 0.8). This value is one of the highest among the studies conducted, as previous studies have mostly reported values within the good (Galdino et al., 2020; Grodberg et al., 2012) and acceptable ranges (Arnold et al., 2016; Irarrázaval et al., 2023; Pagnier & Chaste, 2022; Yang et al., 2023). When Language Pragmatics items were removed, this value increased to the highest level of 0.83. Upon examining item-rest correlations, the lowest value was Language Pragmatics, while the highest value was Interest of Others, in accordance with previous results (Arnold et al., 2016; Galdino et al., 2020; Pagnier & Chaste, 2022). AMSE instructs the observer to skip item 5 if the language item is scored 0 (https://autismmentalstatusexam.com/index). Irarrázaval et al. (2023) reported that they removed item 5 from the internal consistency analysis because it could not be scored by more than half of the participants with ASD (Irarrázaval et al., 2023). Assessing the pragmatic use of language is often not possible in both young children and in many cases where ASD severely affects language development. The majority of children with ASD often scored 0 points on this item, leading to low variability. Additionally, this item includes both feedback and observation-based components. This may explain why it contributed minimally to the internal consistency of the scale. Overall, the results indicated that the internal consistency of the AMSE has been confirmed to varying degrees in different translations and populations.

In this study, the structural validity of the AMSE was examined through EFA and CFA analysis. To the best of our knowledge, no previous research has investigated the factorial structure of the AMSE. The results of both EFA and CFA support a two-factor model, which aligns with the theoretical foundations of the AMSE as conceptualized within the DSM framework. This structure reinforces the distinction between social-communicative and behavioral-sensory aspects of ASD symptomatology. Empirical validation of the factor structure confirms the robustness and psychometric soundness of the instrument in assessing the two core domains of ASD.

To assess the temporal stability of the scale, a test-retest procedure was conducted, and repeated measurements showed excellent correlation (*ICC* = 0.959). This result indicates that the scale is a reliable instrument for distinguishing between the ASD and non-ASD groups over time. When the ICC is 0.9 or higher, a minimum of 6 cases meets the statistical criteria with 90% power and a significance level of *p* = 0.05 (Bujang & Baharum, 2017). Evaluating the test-retest results with a sample of 61 participants, including 20 ASD cases, provides strong evidence for the longitudinal stability of the scale, demonstrating its robust reliability over time. To the best of our knowledge, previous studies have not evaluated its reliability, making this study the first to do so. However, to generalize these results to different samples and populations, replication of the results is warranted.

Additionally, the inter-rater reliability of this tool was excellent (*ICC* = 0.997). In previous studies examining the psychometric properties of the AMSE, inter-rater assessment was also found to be good to excellent (Arnold et al., 2016; Grodberg et al., 2012; Yang et al., 2023). This indicates the success of the test in standardizing observable behaviors and enhancing the validity of the results across different practitioners in diagnosis and scientific research. These results may stem from the fact that studies are generally conducted by expert teams in ASD and the guidelines consist of familiar clinical observations. It is important to repeat these findings, especially among primary healthcare professionals, such as psychologists, child development specialists, and family physicians, to validate the results. Therefore, a careful interpretation is warranted.

In the validation results of this study, AMSE showed an excellent correlation with CARS (*r* = 0.94; *p* < 0.001) in overall sample. Previous studies have found significant and strong relationships between CARS scores and AMSE scores to varying degrees, such as 0.44 (with CARS-2) (Arnold et al., 2016), 0.70 (with CARS) (Pagnier & Chaste, 2022), 0.74 (with CARS) (Yang et al., 2023) and 0.91 (with CARS-BR) (Galdino et al., 2020). The results of this study are similar to those a of study conducted in Brazil, which may be due to methodological similarities and sample size (Galdino et al., 2020). The differences between the study findings may be due to the use of different versions of the CARS and the presence or absence of cases accompanied by developmental delay. ASD exhibits a clinically diverse and heterogeneous phenotype influenced by multiple factors, including age, coexisting conditions such as language impairments and developmental delay, as well as the severity of the ASD itself. Consequently, the AMSE may demonstrate varying psychometric properties across populations characterized by these differing variables. Further studies should be planned by homogenizing groups to achieve the most efficient psychometric measurement tools.

In this study, the correlation between AMSE and CARS was also analyzed separately for ASD and non-ASD groups. The correlation between AMSE and CARS scores was found to be strong in the overall sample (*r* = 0.94); however, when analyzed separately, the correlation remained high in the ASD group (*r* = 0.88) but was weaker in the non-ASD group (*r* = 0.45). This discrepancy may stem from several statistical factors, including between-group differences and variance restrictions. A key explanation for this pattern is the large between-group effect size which likely inflated the overall correlation by amplifying the contrast between ASD and non-ASD participants. Additionally, within the non-ASD group, the restricted range of CARS scores may have contributed to reduced observable variability, consequently weakening the correlation—a phenomenon known as range restriction. It is important to note that the lower correlation within the non-ASD group does not necessarily imply a lack of validity but rather reflects the statistical effects of variance differences and range restriction.

This study was conducted with children referred to our clinic for further assessment owing to the risk of ASD. In the ASD group, the AMSE scores were found to be significantly higher after controlling for age (*p* < 0.001; *Cohen’s d* = 1.40). Similar results were found in studies that included a control group (Cederlund, 2019; Øien et al., 2018; Pagnier & Chaste, 2022). Betz et al. (2019) showed that AMSE could distinguish between children with developmental delay and those with ASD, with significantly higher AMSE scores in children with ASD (Betz et al., 2019). Although it may not be possible to assess the items’ sub-validity and differentiation due to the evaluation of total AMSE scores, the consistent results contribute to the psychometric strength of the tool. These findings demonstrate that AMSE’s diagnostic differentiation remains robust and independent of age effects.

In terms of clinical implications, strong psychometric properties of the AMSE make it a valuable tool for diagnostic assessments conducted by child and adolescent mental health specialists in Türkiye, particularly due to its practicality and efficiency in short examination times. While it effectively supports diagnostic decisions and exclusions in many cases, its use as a screening tool by non-specialists remains unsupported by evidence, despite suggestions from some authors (Galdino et al., 2020; Pagnier & Chaste, 2022). It is important to note that while the AMSE score may suggest an autism diagnosis, it is not sufficient on its own, regardless of case complexity, to establish a definitive diagnosis of autism. The tool was specifically developed for professionals with ASD expertise, and not for primary care providers. In consideration of the objectives of AMSE’s development and the studies conducted, its most appropriate application context appears to be assisting in ASD diagnosis in suspected cases.

The AMSE offers a valuable contribution to ASD assessment by bridging the gap between comprehensive diagnostic tools and parent-report screening measures. It provides a clinician-administered, rapid, and observation-based evaluation of ASD symptoms, integrating both direct behavioral observations and caregiver reports. Unlike self-report tools, the AMSE minimizes reporting bias and facilitates real-time assessment, enhancing diagnostic accuracy. Its structured approach helps standardize clinical observations, promoting consistency while remaining an accessible, practical, and efficient tool for ASD assessment. We recommend that future research should focus on evaluating its reliability across healthcare professionals with varying levels of expertise using standardized methodologies, particularly assessing its feasibility for general practitioners and determining the extent of training required for its effective implementation. In addition, regarding the investigation of AMSE’s item-level functioning, particularly items such as Language Pragmatics, further research is needed to explore its item-level discrimination and ability to detect varying ASD severity levels. We recommend conducting studies with larger and more diverse samples and utilizing advanced analytical methods, such as item response theory analyses, including test information curve, to enhance measurement precision. Finally, the cost-effectiveness of AMSE in non-expert settings remains a concern and should be further investigated. While the AMSE’s online training platform may offer a potential solution, its impact on clinical accuracy and utility has yet to be systematically evaluated.

This study has several strengths, including a relatively large sample size, assessment of validity over time, verification of structural validity through further exploration, and evaluation of psychometric properties in children within the common diagnostic age range for suspected ASD. However, this study has some notable limitations. The primary limitation is that semi-structured diagnostic tools, such as the ADOS-2 and ADI-R, were not utilized, despite the diagnoses being made by a multidisciplinary team. Additionally, the cases were categorized solely into two groups, ASD and non-ASD, and the study did not include an assessment of comorbidities in ASD cases or further diagnostic evaluations in non-ASD cases. This constitutes a limitation in evaluating how the AMSE is affected by factors such as language, intelligence, and social-emotional development. Another limitation of the study might be the test-retest process was conducted by the same clinician who administered the initial diagnostic interview, with a three-week interval between assessments. This may have introduced recall bias, as the clinician’s prior knowledge of the initial evaluation findings could have influenced the AMSE scoring during the second assessment, particularly in parent-reported behaviors.

## Conclusion

AMSE has been shown to be valid in previous studies investigating its psychometric properties in various age groups and different countries, making it a helpful and easily applicable tool for clinicians in the diagnosis of ASD. Our study, conducted with a Turkish sample, demonstrated that the AMSE is a valid and reliable tool with excellent consistency among practitioners. Its widespread use in clinical practice and research as a practical tool has emerged as a significant potential for the early access of individuals with ASD to healthcare and educational services. In particular, the clinical utility of the AMSE may be enhanced by further studies investigating its psychometric properties in different healthcare settings to screen and determine the optimal cut-off values.

## Data Availability

The data that support the findings of this study are available on reasonable request from the corresponding author. The data are not publicly available due to restrictions containing information that could compromise the privacy of research participants.

## Appendix

**Table A1:** Summary of AMSE items (Grodberg et al., 2012)

Score Item	**0**	**1**	**2**
Eye Contact	Consistent	Intermittent	None
Interest in Others	Spontaneous	Passive	None
Pointing Skills	Can point/gesture	Only follows point	None
Language	Complex sentences	Undeveloped sentences, phrases, single words	None
Language Pragmatics	No impairment	Reported impairment	Observed impairment
Repetitive Behaviors	None	Compulsions	Stereotypy
Unusual or Extensive Preoccupations	None	Reported preoccupations	Observed preoccupations
Unusual Sensitivities	None	Reported symptoms	Observed signs
