# Correction to: The Psychometric Properties of Autism Mental Status Examination (AMSE) in Turkish Sample

**DOI:** 10.1007/s10803-025-06813-z

**Published:** 2025-04-01

**Authors:** Yavuz Meral, Alperen Bıkmazer, Abdurrahman Cahid Örengül, Süleyman Çakıroğlu, Esra Altınbilek, Fulya Bakır, Bilgihan Bıkmazer, Ayman Saleh, Vahdet Görmez

**Affiliations:** 1 Istanbul Medeniyet University, School of Medicine, Department of Child and Adolescent Psychiatry, Istanbul, Türkiye; 2 Istanbul University, Istanbul Faculty of Medicine, Department of Child and Adolescent Psychiatry, Istanbul, Türkiye; 3 Altınbaş University, School of Medicine, Child and Adolescent Psychiatry, Istanbul, Türkiye; 4 Göztepe Prof. Dr. Süleyman Yalçın City Hospital, Istanbul, Türkiye; 5 Marmara University Training and Research Hospital, Pediatric Neurology Clinic, Istanbul, Türkiye; 6 George Washington University, Children’s National Hospital, Psychiatry Department, Washington, USA; 7 Hamad Bin Khalifa University, College of Islamic Studies, Doha, Qatar

**Correction to: Journal of Autism and Developmental Disorders (2025)**

10.1007/s10803-025-06761-8

In this article, errors have occurred in the article title, keywords and the sub-section “Autism Mental State Examination (AMSE)” of the Measurements section.

The title was incorrectly given as ‘The Psychometric Properties of Autism Mental State Examination (AMSE) in Turkish Sample’ but should have been ‘The Psychometric Properties of Autism Mental Status Examination (AMSE) in Turkish Sample’.

The keyword ‘Autism mental state examination’ should read as ‘Autism mental Status examination’.

In the ‘Measurements’ section, the sub-heading ‘Autism Mental Status Examination (AMSE)’ was incorrectly written as ‘Autism Mental State Examination (AMSE)’. In this sub section, the sentence ‘total score ranging from 0 to 16’ should read as ‘total score ranging from 0 to 14’.

The original article has been corrected.

